# Tumor marker utility and prognostic relevance of cathepsin B, cathepsin L, urokinase-type plasminogen activator, plasminogen activator inhibitor type-1, CEA and CA 19-9 in colorectal cancer

**DOI:** 10.1186/1471-2407-8-194

**Published:** 2008-07-10

**Authors:** László Herszényi, Fabio Farinati, Romilda Cardin, Gábor István, László D Molnár, István Hritz, Massimo De Paoli, Mario Plebani, Zsolt Tulassay

**Affiliations:** 1 2nd Department of Medicine, Semmelweis University, Budapest Hungarian Academy of Science, Clinical Gastroenterology Research Unit, Budapest, Hungary; 2 2nd Department of Surgery, Semmelweis University, Budapest, Hungary; 3 University of Technology and Economics; SocioMed Ltd., Budapest, Hungary; 4 Department of Surgical & Gastroenterological Sciences (Gastroenterology Unit), University of Padova, Padova, Italy; 5 Department of Central Laboratory, University of Padova, Padova, Italy

## Abstract

**Background:**

Cathepsin B and L (CATB, CATL), urokinase-type plasminogen activator (uPA) and its inhibitor PAI-1 play an important role in colorectal cancer invasion. The tumor marker utility and prognostic relevance of these proteases have not been evaluated in the same experimental setting and compared with that of CEA and CA-19-9.

**Methods:**

Protease, CEA and CA 19-9 serum or plasma levels were determined in 56 patients with colorectal cancer, 25 patients with ulcerative colitis, 26 patients with colorectal adenomas and 35 tumor-free control patients. Protease, CEA, CA 19-9 levels have been determined by ELISA and electrochemiluminescence immunoassay, respectively; their sensitivity, specificity, diagnostic accuracy have been calculated and correlated with clinicopathological staging.

**Results:**

The protease antigen levels were significantly higher in colorectal cancer compared with other groups. Sensitivity of PAI-1 (94%), CATB (82%), uPA (69%), CATL (41%) were higher than those of CEA or CA 19-9 (30% and 18%, respectively). PAI-1, CATB and uPA demonstrated a better accuracy than CEA or CA 19-9. A combination of PAI-1 with CATB or uPA exhibited the highest sensitivity value (98%). High CATB, PAI-1, CEA and CA 19-9 levels correlated with advanced Dukes stages. CATB (*P *= 0.0004), CATL (*P *= 0.02), PAI-1 (*P *= 0.01) and CA 19-9 (*P *= 0.004) had a significant prognostic impact. PAI-1 (*P *= 0.001), CATB (*P *= 0.04) and CA 19-9 (*P *= 0.02) proved as independent prognostic variables.

**Conclusion:**

At the time of clinical detection proteases are more sensitive indicators for colorectal cancer than the commonly used tumor markers. Determinations of CATB, CATL and PAI-1 have a major prognostic impact in patients with colorectal cancer.

## Background

Colorectal cancer (CRC) is the most common gastrointestinal cancer in the Western world and it is an important cause of cancer-related death, tumor stage being generally considered the strongest prognostic factor in CRC [1,2]. Great effort has been dedicated to the search of sensitive and specific markers of the disease and up to now, carcinoembryonic antigen (CEA) and the gastrointestinal cancer-associated carbohydrate antigen (CA 19-9) are the most widely applied markers in gastrointestinal malignancies, *e.g*., CRC or pancreatic cancer [3-7].

Prediction of survival is another feature requested for tumor markers and elevated levels of both CEA and CA 19-9 have also been reported to be associated with poor prognosis in CRC [8-12]. However, because of their low sensitivity, CEA and CA 19-9 seem to be unacceptable both for screening for CRC [3,13,14]. Therefore, there is a need for a search of additional tumor-related antigens, eligible as tumor markers in gastrointestinal malignancies.

Tumor cells have been shown to produce and release several proteolytic enzymes, which are thought to be involved in tumor invasion and metastasis [15]. For instance, it has been observed that cathepsin B (CATB) and cathepsin L (CATL), which are cysteine proteases, the serine protease urokinase-type plasminogen activator (uPA) and its inhibitor type-1 (PAI-1) play a crucial role in this process through the destruction of various elements of the cell-surrounding extracellular matrix [16-21].

Several human solid tumors have been reported to have increased levels of proteolytic enzymes in cancer tissue, strongly suggesting that proteases may be important in tumor invasion and metastasis. With respect to the gastrointestinal tract, we have previously demonstrated that proteolytic enzymes are widely distributed in gastrointestinal tissues, being implicated in processes of gastrointestinal tissue remodelling and angiogenesis [22], may have a role not only in the process of esophageal [23], gastric [24,25] or colorectal cancer invasion [26], but also in the progression of gastrointestinal precancerous changes into cancer [27].

Cathepsins and components of the plasminogen activator and inhibitor system have been demonstrated in various malignant tissues, *e.g*., breast cancer [28-30], lung cancer [31,32], head and neck cancer [33], ovarian cancer [34], gastric cancer [35-38] or CRC [39-45] and might therefore be useful as a diagnostic tool.

With respect to the gastrointestinal tract, several studies, along with our own, have pointed to the prognostic value of proteases for survival, for instance, in gastric cancer [24,25,38,46,47] and CRC [26,40,43,48-50].

A number of studies have demonstrated an elevation of serum or plasma protease levels in patients suffering from CRC [51-55], however, to our knowledge, the tumor marker utility of CATB, CATL, uPA and PAI-1 has not been evaluated in the same experimental setting, and compared with that of the most commonly used gastrointestinal tumor markers, such as CEA and CA-19-9.

Therefore, the objective of the present study was to assess the possible clinical relevance of serum CATB, CATL and plasma uPA, PAI-1 antigen levels in the same CRC patients, compare them with the already established serum markers CEA and CA 19-9, and to evaluate any correlation between these parameters and clinicopathological and prognostic staging of CRC.

## Methods

The study comprised 56 patients with CRC, who underwent colorectal resection, 29 males and 27 females, mean age 65.4 ± 12.8 years (range 39–86 years) and 35 tumor-free control patients (controls) with negative gastroscopy and colonoscopy, 12 males and 23 females, mean age 46.3 ± 13.4 years (range 24–85 years). For further comparison we also investigated 25 patients with ulcerative colitis (UC) without dysplasia as confirmed by colonoscopy and biopsy, 11 males and 14 females, mean age: 31.7 ± 6.6 years (range 22–48 years), and 26 patients with colorectal adenoma confirmed by histology after endoscopic polypectomy (samples consisted of 16 tubular adenomas with low grade dysplasia and 10 tubulovillous adenomas with high grade dysplasia), 17 males and 9 females, mean age 57.8 ± 6.9 years (range 47–72 years). Informed consent was obtained from all patients. The study was approved by the Ethical Committee of Semmelweis University Budapest and Padova University.

In all instances, this was a first diagnosis of CRC, and no recurrences were taken into consideration. Patients who were undergoing chemotherapy, radiotherapy, or any other adjuvant therapy for CRC, or were expected to undergo such therapies within the study period were not included.

Clinical data of the patients and histology of tumors were registered accurately. Pathologic staging was obtained for the presence (n= 37) or absence (n = 19) of lymph node and/or distant metastases, and for differentiation (well differentiated, G1 (n = 17); moderately differentiated, G2 (n = 30); or poorly differentiated, G3 (n = 9). Finally, the tumors were also subgrouped according to their tumor location (colon cancer, n = 38; rectal cancer, n = 18). The tumors were histologically classified according to Dukes classification, as modified by Turnbull *et al*. [56]. Dukes stage A tumors were confined to the bowel wall (n = 7); Dukes stage B tumors have spread beyond the wall without involving lymph nodes (n = 12); Dukes stage C were associated with regional lymph node metastases (n = 23); and finally, Dukes stage D tumors were associated with distant metastases (n = 14).

### Blood collection

Serum and plasma samples were collected from patients with CRC at the time of clinical tumor detection. Blood samples were collected from resting patients after a 12 h fasting between 8:00 and 10:00 a.m. to avoid possible influences of circadian variations on the fibrinolytic system. Blood was drawn from a cubital vein with minimal venous occlusion directly into plastic tubes prepared with sodium citrate (0.1 M final concentration) as an anticoagulant to avoid platelet activation and platelet contamination. Plasma was obtained by centrifugation at 3000 × g for 15 min at 4°C, harvested and snap-frozen. The samples were stored at -70°C until analysis.

### Determination of established tumor markers and proteases

Serum CEA (carcinoembryonic antigen electrochemiluminescence immunoassay "ECLIA", Cobas^®^, Roche, Diagnostics, Mannheim, Germany; cut-off limit, 4.0 ng/ml) and serum CA 19-9 (carbohydrate electrochemiluminescence immunoassay "ECLIA", Cobas^®^, Roche, Diagnostics, Mannheim, Germany; cut-off limit, 37.0 ng/ml) were determined by commercially available test kits and monoclonal antibodies. Cut-off limits were taken as recommended by the manufacturers.

The assays for CATB, CATL, uPA and PAI-1 have been published elsewhere [22,26]. Antigen levels were measured by using the enzyme-linked immunoassay (ELISA) method as follows: briefly, cathepsin immunoassay is a solid-phase ELISA based on the sandwich principle (BiAss, Diesen, Germany). Absolute quantities of CATB and CATL antigens in the serum samples were calculated from a 7-point standard curve of CATB and CATL (0–250 ng/ml). The lowest detectable concentrations were estimated at ≅ 1 ng/ml.

The uPA antigen was determined by using the TintElize uPA-ELISA (Biopool, Umea, Sweden). The amount of uPA antigen in the plasma samples was calculated from a 6-point standard curve of uPA (0–4 ng/ml). The detection limit was ≅ 0.1 ng/ml for uPA.

PAI-1 antigen quantification was performed by using the Asserachrom PAI-1 ELISA (Diagnostica Stago, Asniéres-sur-Seine, France). Absolute quantities of PAI-1 antigen in the plasma samples were calculated from a 7-point standard curve of PAI-1 (0–250 ng/ml). The detection limit was ≅ 0.5 ng/ml for PAI-1.

### Statistics

Due to the high standard deviations of some of the series, results were expressed as median levels and, after evaluation by using the Kolmogorov-Smirnov test, differences between groups were tested statistically by using the Mann-Whitney U test and the Kruskall-Wallis analysis of variance, where applicable. Spearman rank correlation test was performed to evaluate the correlation between CATB, CATL, uPA, PAI-1, CEA and CA 19-9. Differences were considered significant with *P *< 0.05. The receiver operating characteristics (ROC) curves were used to determine the optimal cut-off values (with the Youden J test for overall accuracy). The areas under curve (AUCs) for all investigated biomarkers were determined according to ROC curves. The discriminative power of each biomarker for the diagnostic accuracy was tested by Fisher's exact test.

The association of proteases and CEA, CA 19-9 and survival was tested using their median values in the group of CRC. The Kaplan-Meier method was used to estimate survival probabilities, and the log-rank test was used to test equality of strata. Group-oriented curves for survival were calculated according to the Kaplan-Meier method for CATB, CATL, uPA and PAI-1 antigen levels; CEA and CA 19-9; Dukes classification; grade; tumor location; age and gender. The Cox proportional hazards model was applied for multivariate analysis. Variables were included in the multivariate analysis only if the *P *value was less than 0.05 in the univariate analysis. The SAS software package (SAS Institute, Cary, North Carolina) was used to perform statistical analyses.

## Results

Serum and plasma concentrations for CATB, CATL, uPA, PAI-1, CEA and CA 19-9 in patients with CRC, UC, colorectal adenoma and controls, expressed in ng/ml, are shown in Table 1. Significantly higher CATB, CATL, uPA and PAI-1 antigen concentrations were observed in CRC patients compared with controls, patients with UC or colorectal adenomas. No statistically significant differences were seen with respect to CEA and CA 19-9 levels. Antigen levels of CATB, CATL and PAI-1 were significantly higher in blood samples from patients with colorectal adenomas than from controls. CATB, CATL, uPA and PAI-1 showed a trend towards increase in patients with UC compared with controls, but the differences were statistically not significant (Table 1).

**Table 1:** Proteolytic enzymes, CEA and CA 19-9 in patients with colorectal cancer, ulcerative colitis, adenoma and controls.

	**CATB**	**CATL**	**uPA**	**PAI-1**	**CEA**	**CA 19-9**
**Colorectal cancer **(n = 56)	8.75*§ (2.4–39.3)	1.10*§ (1.0–35.3)	0.29*§ (0.1–0.79)	52.45*§ (13.5–138.6)	2.40 (0.4–235.0)	9.15 (1.0–540.0)
**Ulcerative colitis** (n = 25)	4.37 (2.8–7.2)	1.10 (1.0–1.7)	0.20 (0.18–0.23)	12.60 (8.8–17.6)	3.15 (0.8–11.5)	9.00 (4.5–32.0)
**Colorectal adenoma **(n = 26)	4.45# (4.2–10.3)	1.25# (1.0–6.5)	0.20 (0.16–0.53)	14.05# (10.5–65.5)	3.55 (0.7–5.7)	10.27 (3.7–41.0)
**Controls** (n = 35)	3.80 (1.4–5.8)	1.00 (1.0–2.1)	0.19 (0.16–0.27)	10.70 (1.87–23.8)	2.90 (0.8–18.9)	8.90 (1.27–47.4)
Kruskall-Wallis analysis of variance (*P *value)	*P *< 0.0001	*P *< 0.0001	*P *< 0.0001	*P *< 0.0001	*P *= NS	*P *= NS

Cathepsin B, cathepsin L, urokinase-type plasminogen activator, plasminogen activator inhibitor type-1, CEA and CA 19-9 levels in patients with colorectal cancer (n = 56), ulcerative colitis (n = 25), colorectal adenoma (n = 26) and controls (n = 35) expressed in ng/ml (median values and range)

*Statistics*:

* Colorectal cancer: *P *< 0.001 *vs *Controls

§ Colorectal cancer: *P *< 0.05 *vs *Ulcerative colitis and Colorectal adenoma

# Colorectal adenoma: *P *< 0.001 *vs *Controls

*Abbreviations*: CATB: cathepsin B; CATL: cathepsin L; uPA: urokinase-type plasminogen activator, PAI-1: plasminogen activator inhibitor type-1; CEA: carcinoembryonic antigen; CA 19-9: carbohydrate antigen 19-9; NS: not significant

With respect to Dukes classification, CATB, PAI-1, CEA and CA 19-9 showed the highest antigen concentrations in patients with Dukes stage D tumors (Table 2).

**Table 2:** Proteolytic enzymes, CEA and CA 19-9 in correlation with Dukes classification.

**Stage**	**CATB**	**CATL**	**uPA**	**PAI-1**	**CEA**	**CA 19-9**
**DUKES A **(n = 7)	4.50 (2.4–37.3)	1.10 (1.0–35.3)	0.30 (0.2–0.62)	32.81 (17.2–66.7)	3.00 (0.6–3.4)	6.80 (3.0–11.5)
**DUKES B **(n = 12)	6.75 (3.2–23.8)	1.00 (1.0–12.3)	0.29 (0.1–0.79)	39.09 (13.5–108.1)	2.00 (0.6–21.1)	7.65 (1.0–35.0)
**DUKES C **(n = 23)	8.60 (3.6–35.7)	1.10 (1.0–34.1)	0.26 (0.1–0.79)	52.54 (13.8–126.4)	2.20 (0.4–235.0)	9.20 (1.0–78.6)
**DUKES D **(n = 14)	24.25*§ (9.2–39.3)	4.55 (1.0–35.2)	0.29 (0.12–0.42)	82.03** (38.7–138.6)	8.30# (1.9–74.6)	35.85** (4.6–540.0)
Kruskall-Wallis analysis of variance (*P *value)	*P *= 0.0002	*P *= NS	*P *= NS	*P *= 0.01	*P *= 0.003	*P *= 0.001

Cathepsin B, cathepsin L, urokinase-type plasminogen activator, plasminogen activator inhibitor type-1, CEA and CA 19-9 levels in association with Dukes classification of colorectal cancer expressed in ng/ml (median values and range)

*Statistics*:

*P < 0.01, Dukes D *vs *Dukes A and B

§*P *< 0.05, Dukes D *vs *Dukes C

**P < 0.05, Dukes D *vs *Dukes A, B and C

#*P *< 0.01, Dukes D *vs *Dukes A, B and C

*Abbreviations*: DUKES A: Dukes stage A; DUKES B: Dukes stage B; DUKES C: Dukes stage C; DUKES D: Dukes stage D; CATB: cathepsin B; CATL: cathepsin L; uPA: urokinase-type plasminogen activator, PAI-1: plasminogen activator inhibitor type-1; CEA: carcinoembryonic antigen; CA 19-9: carbohydrate antigen 19-9; NS: not significant

Antigen levels of CATB, CATL, uPA, PAI-1 and CEA, CA 19-9 levels showed a trend toward higher levels in patients with colon cancer compared to patients with rectal cancer, but the differences were not statistically significant (data not shown). No statistically significant changes were observed in association with tumor grade, age or gender (data not shown).

The receiver operating characteristics (ROC) curves were used to determine the optimal cut-off values (with the Youden J test for overall accuracy). The optimal cut-off value for CATB was 4.60 ng/ml (Youden *J *= 0.68), the cut-off for CATL was placed at 1.12 ng/ml (Youden *J *= 0.25). The optimal selected cut-off values for uPA and PAI-1 were 0.21 ng/ml (Youden *J *= 0.53) and 18.90 ng/ml (Youden *J *= 0.75), respectively. Cut-off limits for CEA (4.0 ng/ml) and CA 19-9 (37.0 ng/ml) were taken as recommended by the manufacturers.

Sensitivity was calculated as the percentage of individuals in the tumor groups (test-positive tumor patients) who showed concentrations of tumor markers above the respective cut-off limits. Specificity was calculated as the percentage of test-negative, non-tumor individuals, which could be either healthy individuals (controls), those with UC or patients with colorectal adenomas.

When proteases, CEA and CA 19-9 were used as single markers, considering the above mentioned optimal cut-off values, sensitivity of PAI-1 (94%), CATB (82%), uPA (69%) and CATL (41%) were more indicative for CRC than CEA or CA 19-9 (30% and 18%, respectively). Specificity of CATB (88%) and PAI-1 (84%) was in the same range than that of the established markers (CA 19-9: 93%, CEA: 89%) (Table 3).

**Table 3:** Diagnostic accuracy of proteolytic enzymes, CEA and CA 19-9 in colorectal cancer.

	**CATB**	**CATL**	**uPA**	**PAI-1**	**CEA**	**CA 19-9**
***Cut-off***	(4.60)	(1.12)	(0.21)	(18.90)	(4.0)	(37.0)
**Sensitivity **(%)	82	41	69	94	30	18
**Specificity **(%)	88	80	82	84	89	93
**PPV **(%)	82	58	72	79	65	62
**PPV-Positive Prevalence **(%)	43	19	33	40	26	23
**NPV **(%)	88	68	80	96	66	63
**NPV-Negative Prevalence **(%)	27	7	19	35	5	2
**Accuracy **(%)	86	65	77	88	66	63
***Fisher's Exact Test***	***P ***< 0.0001	***P ***= 0.008	***P ***< 0.0001	***P ***< 0.0001	***P ***= 0.004	***P ***= 0.06

Sensitivity, specificity, positive predictive value, negative predictive value and accuracy of cathepsin B, cathepsin L, urokinase-type plasminogen activator, plasminogen activator inhibitor type-1, CEA and CA 19-9 in colorectal cancer

*Abbreviations*: PPV: Positive predictive value; NPV: Negative predictive value; CATB: cathepsin B; CATL: cathepsin L; uPA: urokinase-type plasminogen activator, PAI-1: plasminogen activator inhibitor type-1; CEA: carcinoembryonic antigen; CA 19-9: carbohydrate antigen 19-9; Cut-off: cut-off values expressed in ng/ml; Positive Prevalence: the percentage of tumor patients in all patient groups investigated, i.e. 56/142 = 39%; Negative Prevalence: the percentage of non-tumor individuals in all patient groups investigated, i.e. 86/142 = 61%; PPV-Positive Prevalence: the difference between PPV and positive prevalence; NPV-Negative Prevalence: the difference between NPV and negative prevalence

PPV (probability of detection a CRC by a positive test) and NPV values (probability of exclusion of a CRC suspicion by a negative test) are post-test probabilities and thus strongly dependent on the pre-test probability (positive prevalence) reflecting the percentage of tumor patients in all patient groups investigated, i.e. 56/142 = 39%, and negative prevalence reflecting the percentage of non-tumor individuals in all patient groups investigated, i.e. 86/142 = 61%. The comparison of positive prevalence with PPV and negative prevalence with NPV allows to judge the utility of PPV and NPV values in relation to a probability by chance, i.e. they should differ from the prevalence by at least 20%. According to this, the PPV values were of relevance for CATB, uPA, PAI-1, CEA and CA 19-9, and less for CATL; inversely, the NPV values were of relevance for CATB, PAI-1, uPA, but not at all for CATL, CEA and CA 19-9 (Table 3).

PAI-1, CATB and uPA demonstrated a better diagnostic accuracy than CEA, CATL or CA 19-9, PAI-1 showed the highest accuracy (Table 3).

**Figure 1:**
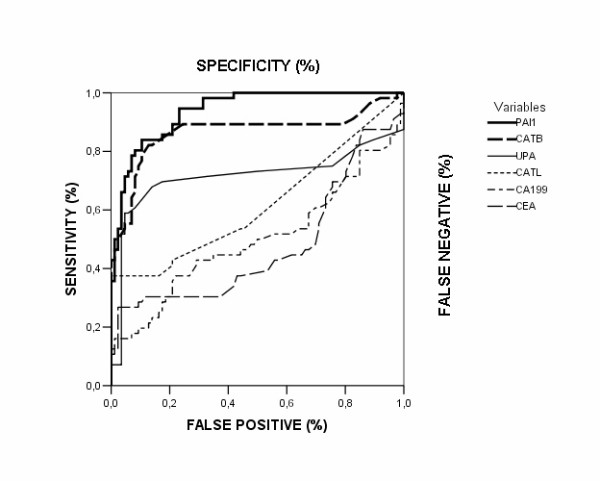
**ROC curves for proteolytic enzymes, CEA and CA 19-9 in patients with colorectal cancer**. The clinical relevance of cathepsin B (CATB), cathepsin L (CATL), urokinase-type plasminogen activator (uPA), plasminogen activator inhibitor type-1 (PAI-1), CEA and CA 19-9 was compared by establishing ROC curves. The ROC curves of PAI-1 and CATB were located closer to the theoretical 100% sensitivity and specificity values than the ROC curves of other investigated markers.

The clinical relevance of the different markers is demonstrated by ROC curves. The ROC curves of PAI-1 and CATB were located closer to the theoretical 100% sensitivity and specificity values than the ROC curves of other investigated markers (Figure 1).

Table 4 summarizes the areas under curve (AUCs) for all investigated biomarkers. Again, PAI-1 and CATB demonstrated the highest accuracy and the best discriminative power.

**Table 4:** The areas under curve for proteolytic enzymes, CEA and CA 19-9 according to ROC curves.

**Variable**	**AUC**	**Lower limit**	**Upper limit**
**PAI-1**	0.9385	0.9025	0.9744
**CATB**	0.8665	0.7934	0.9397
**uPA**	0.7143	0.6091	0.8196
**CATL**	0.6173	0.5158	0.7188
**CA 19-9**	0.4990	0.3937	0.6043
**CEA**	0.4748	0.3690	0.5807

Determination of areas under curve for cathepsin B, cathepsin L, urokinase-type plasminogen activator, plasminogen activator inhibitor type-1, CEA and CA 19-9 according to ROC curves allowing to evaluate their accuracy and different discriminative power

*Abbreviations*: AUC: area under curve; CATB: cathepsin B; CATL: cathepsin L; uPA: urokinase-type plasminogen activator, PAI-1: plasminogen activator inhibitor type-1; CEA: carcinoembryonic antigen; CA 19-9: carbohydrate antigen 19-9; ROC: receiver operating characteristics

Sensitivity and specificity values during multiparametric tumor marker analysis are given in Table 5. When two markers were determined in identical blood samples, combined sensitivity values disclosed the superiority of the combination of PAI-1 together with CATB or uPA (both markers correctly test-positive for tumor patients in 78% and 64%, respectively; one of two markers correctly test-positive in the tumor group in 98%) as compared to the combinations of all other markers, including proteases with CEA or CA 19-9, or CEA with CA 19-9. The sensitivity of CEA or CA 19-9 in combination with a protease antigen level was more indicative for CRC than CEA or CA 19-9 alone (when one of two markers was correctly test-positive). The combined use of three markers (one protease in combination with CEA and CA 19-9) did not lead to a further increase in sensitivity.

**Table 5:** Multiparametric tumor marker analysis in colorectal cancer.

	**Colorectal cancer**			
	**Sensitivity**		**Specificity**	

**Two markers**	***Both positive***	***Either positive***	***Both negative***	***Either negative***

PAI-1 + CATB	78%	98%	73%	98%
PAI-1 + uPA	64%	98%	67%	98%
PAI-1 + CATL	39%	96%	67%	96%
CATB + uPA	55%	95%	79%	87%
CATB + CATL	41%	84%	75%	94%
CATL + uPA	30%	80%	72%	90%
				
PAI-1 + CEA	30%	96%	73%	98%
CATB + CEA	28%	84%	83%	95%
uPA + CEA	20%	80%	76%	95%
CATL + CEA	20%	52%	75%	94%
				
PAI-1 + CA 19-9	18%	96%	77%	98%
CATB + CA 19-9	16%	84%	86%	97%
uPA + CA 19-9	14%	73%	80%	95%
CATL + CA 19-9	11%	48%	78%	95%
CEA + CA 19-9	16%	32%	86%	97%

				

**Three markers**	***All positive***	***Either positive***	***All negative***	***Either negative***

PAI-1 +CEA +CA 19-9	16%	96%	71%	98%
CATB +CEA +CA 19-9	14%	84%	80%	97%
uPA +CEA +CA 19-9	12%	80%	74%	97%
CATL +CEA +CA 19-9	9%	52%	74%	97%

Sensitivity and specificity values of cathepsin B, cathepsin L, urokinase-type plasminogen activator, plasminogen activator inhibitor type-1, CEA and CA 19-9 during multiparametric tumor marker analysis in colorectal cancer (n = 56)

CATB: cathepsin B; CATL: cathepsin L; uPA: urokinase-type plasminogen activator, PAI-1: plasminogen activator inhibitor type-1; CEA: carcinoembryonic antigen; CA 19-9: carbohydrate antigen 19-9;

Both: both markers correctly positive or negative; All: all three markers correctly positive or negative; Either: one of two/or three markers correctly positive or negative

The specificity of CEA or CA 19-9 in combination with a protease antigen level or the combination of CEA with CA 19-9 was more indicative for correctly negative patients than CEA or CA 19-9 alone (when one of two markers was correctly test-negative for non-tumor patients). When two protease levels were considered, combined specificity values (one of two markers correctly test-negative for non-tumor group) were also higher than specificity of CATB, CATL, uPA or PAI-1 as a single marker. The combined use of three markers (one protease in combination with CEA and CA 19-9) did not lead to a further increase in specificity (Table 5).

Table 6 demonstrates associations between proteases and CEA/CA 19-9, determined in identical blood samples obtained from CRC patients. When correlation analysis was assessed between proteases, CATB significantly correlated with CATL, uPA and PAI-1. Significant correlations were also found between the antigen levels of PAI-1 and CATL, and finally PAI-1 and uPA. CEA and CA 19-9 also significantly correlated in the group of CRC. When Spearman rank correlation test was performed in CRC between CEA, CA 19-9 and proteases, both CEA and CA 19-9 significantly correlated with CATB and PAI-1.

**Table 6:** Correlation analysis of proteolytic enzymes, CEA and CA 19-9 in colorectal cancer.

	**Colorectal cancer**	
	***P *value**	**(correlation coefficient)**

**CATB/CATL**	*P *= 0.0001	(rS = 0.67)
**CATB/uPA**	*P *= 0.03	(rS = 0.28)
**CATB/PAI-1**	*P *= 0.04	(rS = 0.27)
**CATL/uPA**	*P *= 0.11	(rS = 0.21)
**PAI-1/CATL**	*P *= 0.03	(rS = 0.27)
**PAI-1/uPA**	*P *= 0.001	(rS = 0.41)
		
**CEA/CA 19-9**	*P *= 0.0003	(rS = 0.46)
**CEA/CATB**	*P *= 0.002	(rS = 0.40)
**CEA/CATL**	*P *= 0.06	(rS = 0.25)
**CEA/uPA**	*P *= 0.70	(rS = 0.05)
**CEA/PAI-1**	*P *= 0.002	(rS = 0.40)
**CA 19-9/CATB**	*P *= 0.005	(rS = 0.36)
**CA 19-9/CATL**	*P *= 0.33	(rS = 0.13)
**CA 19-9/uPA**	*P *= 0.46	(rS = 0.10)
**CA 19-9/PAI-1**	*P *= 0.005	(rS = 0.36)

Correlation of cathepsin B, cathepsin L, urokinase-type plasminogen activator, plasminogen activator inhibitor type-1, CEA and CA 19-9 in patients with colorectal cancer (n = 56)

*Abbreviations*: CATB: cathepsin B; CATL: cathepsin L; uPA: urokinase-type plasminogen activator, PAI-1: plasminogen activator inhibitor type-1; CEA: carcinoembryonic antigen; CA 19-9: carbohydrate antigen 19-9; rS: Spearman rank correlation coefficient

The CRC patients were enrolled in a follow-up protocol. The follow-up ended at the event of death or, when the patient was still alive, at the last follow-up date. Patients were followed either directly or through their attending physicians. Thirty-five patients (62.5%) died of tumor recurrence. Their median survival was 32 months (95% CL, 23–38; range, 7–78 months). At the end of follow-up period, 21 patients (37.5%) were still alive; their median follow-up was 86 months (95% CL, 85–91; range 76–96 months). The median survival time calculated for all patients was 45 months (95% CL, 43–61; range 7–96 months). The median survival for the subgroup of patients who underwent curative resection (Dukes A-C) was 77 months (95%CL, 56–73, range 7–96 months), whereas it was 14 months in the remaining group of patients with Dukes D tumors (95%CL, 11–19; range, 8–36 months; *P *< 0.001).

No statistically significant difference was observed in association with tumor location (median survival period for patients with colon cancer and rectal cancer: 43 months, 95%CL, 40–62; range, 8–96 months, and 55 months, 95%CL, 37–70, range, 7–94 months, respectively).

The association of proteolytic enzymes and CEA, CEA-19 and survival was tested using their median values in the group of CRC (CATB: 8.75 ng/ml; CATL: 1.1 ng/ml; uPA: 0.29 ng/ml; PAI-1: 52.45 ng/ml; CEA: 2.40 ng/ml; CA 19-9: 9.15 ng/ml).

**Figure 2:**
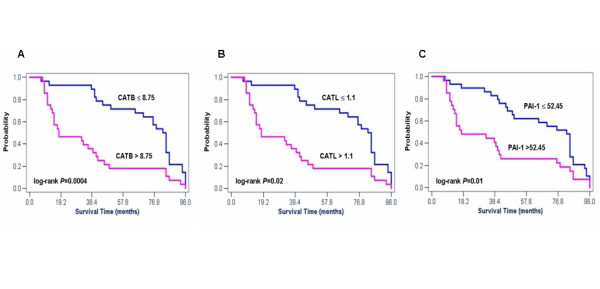
**Survival curves stratified by cathepsin B, cathepsin L and plasminogen activator inhibitor type-1 in colorectal cancer**. The association of preoperative serum cathepsin B (CATB), cathepsin L (CATL) and plasma plasminogen activator inhibitor type-1 (PAI-1) antigen levels and overall survival in patients with primary colorectal carcinoma (CRC) (n = 56). By using the median values of CATB, CATL and PAI-1 the patients were divided into two groups. High antigen levels identified patients with shorter survival and those who were at higher risk of death. Group-oriented curves for survival were calculated according to the Kaplan-Meier method. The *P *values are shown in the figure. **A**. CATB in sera of patients with CRC (CATB median as a cut-off value, 8.75 ng/ml). **B**. CATL in sera of patients with CRC (CATL median as a cut-off value, 1.1 ng/ml). **C**. PAI-1 in plasma of patients with CRC (PAI-1 median as a cut-off value, 52.45 ng/ml).

In univariate survival analysis, CATB antigen levels (*P *= 0.0004) (Figure 2A), CATL antigen levels (*P *= 0.02) (Figure 2B), PAI-1 antigen levels (*P *= 0.01) (Figure 2C), CA 19-9 (*P *= 0.004), and Dukes classification (*P *< 0.0001) were significantly correlated with survival. No significant correlation was observed with respect to uPA antigen levels, CEA, tumor grade, tumor location, age and gender. To determine the independent value of these prognostic factors, multivariate analysis was performed. Variables were included in the multivariate analysis only if the *P *value was less than 0.05 in the univariate analysis. In the multivariate statistical analysis all parameters – including Dukes classification, PAI-1, CATB, CATL and CA 19-9 – were tested in one model. According to this, significant independent prognostic information was obtained from Dukes classification (*P *= 0.004), PAI-1 (*P *= 0.001), CATB (*P *= 0.04) and CA 19-9 (*P *= 0.02), but not from CATL. Analysed as continuous variables in the Cox model, PAI-1 (HR 2.22, *P *= 0.001), CATB (HR 2.09, *P *= 0.04) and CA 19-9 (HR 1.98, *P *= 0.02) were found to be Dukes-independent prognostic factors (Table 7).

**Table 7:** Univariate and multivariate analysis of survival in patients with colorectal cancer.

	**Univariate analysis**	**Multivariate analysis**		
	**Log-Rank Test**	**Cox Proportional Hazards Model**		

**Variable**	***P *value**	***P *value**	**HR**	**(95% CL)**

**Dukes**	< 0.0001	0.004	1.88	(1.22–2.89)
**PAI-1**	0.012	0.001	2.22	(1.21–4.06)
**CATB**	0.0004	0.042	2.09	(1.02–4.26)
**CATL**	0.021	0.639	1.17	(0.60–2.29)
**CA 19-9**	0.004	0.024	1.98	(1.09–3.58)
				
**uPA**	0.369			
**CEA**	0.159			
**Grade**	0.420			
**Location**	0.566			
**Age**	0.247			
**Gender**	0.122			

Univariate analysis including Dukes classification, cathepsin B, cathepsin L, urokinase-type plasminogen activator, plasminogen activator inhibitor type-1, CEA, CA 19-9, tumor grade, tumor location, age, and gender. Variables were included in the multivariate analysis only if the *P *value was less than 0.05 in the univariate analysis. In the multivariate statistical analysis all parameters -including Dukes classification, PAI-1, CATB, CATL and CA 19-9 – were tested in one model.

*Abbreviations*: Dukes: Dukes classification; CATB: cathepsin B; CATL: cathepsin L; uPA: urokinase-type plasminogen activator, PAI-1: plasminogen activator inhibitor type-1; CEA: carcinoembryonic antigen; CA 19-9: carbohydrate antigen 19-9; Grade: tumor grade (differentiation); Location: tumor location; HR: Hazard ratio; 95% CL: 95% Hazard ratio confidence limits

## Discussion

Among several tumor markers, that are suggested to correlate with the presence and prognosis of CRC, CEA and CA 19-9 are the most widely accepted [3-7]. However, the rather low sensitivity of serum CEA or CA 19-9, their secretion rates from individual tumors and nonspecific elevations reduce their tumor marker utility and indicate the need for additional more reliable markers for CRC.

Proteolytic mechanisms, such as those that depend on cysteine (CATB, CATL) and serine proteases (uPA, PAI-1) are recognized as crucial factors in tumor invasion and metastasis. Cysteine proteases (CATB, CATL) play an important role in this process through the destruction of various elements of cell-surrounding extracellular matrix, whereas uPA appears to promote invasion through a plasmin-mediated degradation of extracellular matrix proteins. Active uPA catalyses the conversion from plasminogen to plasmin, that is a potent activator of several metalloproteinase proenzymes, such as prostromelysin, procollagenase, and progelatinase. Beyond its direct proteolytic capacity, CATB has been shown also to activate the pro-urokinase-type plasminogen activator (pro-uPA). PAI-1 under normal physiologic conditions inhibits uPA by forming a covalent enzyme-inhibitor complex. However, the exact role of PAI-1 in tumor biology is not well established. It has been suggested that PAI-1 may represent a specific protein of transformed malignant tissue and it has also been claimed that PAI-1 may serve to protect cancer tissue against the proteolytic degradation triggered by the tumor on surrounding normal tissue. Furthermore, the inhibitor has a role in angiogenesis, which has an important part to play in tumor spread [15-21].

Several studies have suggested the potential impact of proteases as tumor markers in CRC [51-55]. Given the lack in the literature for a comparison of the tumor marker utility and possible prognostic relevance of cathepsins (CATB, CATL) and the uPA/PAI-1 system in the same experimental setting, in the current study, we surveyed the behavior of CATB, CATL, uPA, PAI-1 in CRC and compared with commonly used gastrointestinal tumor markers CEA and CA 19-9, and then evaluated any correlation between these parameters and the clinicopathological staging of CRC.

In our study, antigen levels of proteases were measured by using ELISA method. Most ELISAs used to measure uPA and PAI-1 levels generally are unable to distinguish between the different forms of uPA and PAI-1; thus, the overall ELISA signal results from a mixture of substances (e.g., pro-, active, and complexed forms of the analyte). Therefore, it has been proposed that the levels of uPA:PAI-1 complex may provide additional valuable predictive information. Indeed, it has been demonstrated by Manders *et al*. that increased uPA:PAI-1 complex levels, like uPA and PAI-1 levels separately, were significantly associated with poor overall survival, moreover, the expression of uPA:PAI-1 complex independently predicted the efficacy of adjuvant chemotherapy in patients with primary breast cancer [29].

We confirm the previous observations that preoperative serum CATB [51,53,55], plasma uPA [52] and PAI-1 [54] concentrations are significantly higher in CRC than those found in control non-cancer patients. In addition, with respect to cathepsins, we demonstrated that not only CATB but also CATL is elevated in sera of CRC patients. Interestingly, no statistically significant differences were seen in association with CEA and CA 19-9.

We demonstrate for the first time that antigen levels of CATB, CATL and PAI-1 were significantly higher in blood samples from patients with colorectal adenomas compared to the controls. Thus our results with previous results obtained in colorectal tissues [42,57] confirm that CATB, CATL and PAI-1 may be involved in the progression from premalignant colorectal adenoma into CRC.

When proteases, CEA and CA 19-9 were used as single markers, we found that sensitivity of PAI-1 (94%), CATB (82%), uPA (69%) and CATL (41%) were more indicative for CRC than CEA or CA 19-9 (30% and 18%, respectively). Our data are in agreement with observations of Huber *et al*. [52]. They have reported that sensitivity of uPA was superior to that of the established markers (75.5% *vs*. 51.5% of both CEA and CA 19-9). Specificity of CATB and PAI-1 were in the same range than that of CA 19-9 or CEA. PAI-1, CATB and uPA demonstrated a better accuracy than CEA or CA 19-9, PAI-1 showing the highest accuracy.

It has been suggested that a combined use of different tumor-associated antigens might be of better clinical value for the detection and follow-up of various cancers. The simultaneous determination of several markers led to a greater sensitivity in our group of CRC patients: PAI-1 combined with CATB or UPA was superior compared to the combination of all other markers, including proteases with CEA or CA 19-9. In addition, the sensitivity of CEA or CA 19-9 in combination with a protease antigen level was more indicative for CRC than CEA or CA 19-9 alone. Furthermore, during the multiparametric tumor marker analysis, the combined use of two markers in all combinations led to a further increase in specificity.

This observation was confirmed by the data we obtained by examining the correlation of the investigated parameters with Dukes staging, an established predictor of prognosis. While CATL and uPA did not show any correlation with the stage, CATB, PAI-1, CEA and CA 19-9 did show a significant increase in patients with advanced stage.

Increased pre-operative serum levels of CEA and CA 19-9 have been already shown to correlate with shorter disease free and overall survival [8-12] It has been also previously reported that higher serum levels of CATB [51,53,55] and plasma levels of PAI-1 [54] are correlated with advanced tumor stage and shorter survival in CRC. According to our experience, in a univariate survival analysis not only CATB and PAI-1, but also CATL antigen levels were significant in prediction of survival. High serum CATB, CATL and plasma PAI-1 antigen levels indeed identified patients with shorter survival and those who were at higher risk of death. In addition, PAI-1 and CATB proved as independent predictor variables in a multivariate statistical analysis. With respect to the commonly used tumor markers, only CA 19-9 correlated significantly with survival and was found to be independent prognostic factor. In our series no significant correlation was observed in association with respect to uPA antigen levels, CEA, tumor grade, tumor location, age and gender.

The data in the literature and our own on patient's survival suggest that serum CATB, CATL and plasma PAI-1 levels might be more useful than the traditionally used tumor markers. Our results are in full agreement with recent guidelines regarding the use of CEA or CA 19-9 in CRC. According to these guideline recommendations, inadequate sensitivity severely limits the value of CEA for the diagnosis of CRC, therefore CEA should not be used to screen for early CRC. Further, data are insufficient to support the use of CEA to determine whether to treat a patient with adjuvant chemotherapy. On the other hand, CEA is the marker of choice for monitoring metastatic CRC during systemic therapy. With respect to CA 19-9, although preoperative elevated levels may provide prognostic information, present data are insufficient to recommend CA 19-9 for screening, diagnosis, surveillance or monitoring treatment of patients with CRC [58,59].

We report for the first time that serum CATB antigen levels significantly correlate with plasma uPA and PAI-1 levels in CRC, and a significant correlation is also found between the antigen levels of CATL and PAI-1. This confirms previous data obtained in gastrointestinal cancerous tissues on a concomitant activation of these systems [21,24,26,27], while the simultaneous up-regulation of cysteine and serine proteases in CRC strongly confirms the role of cathepsins and the uPA/PAI-1 system in the biology of CRC.

## Conclusion

In summary, our data provide evidence for possible clinical application of the determination of CATB, CATL, uPA and PAI-1 in addition to CEA and CA 19-9 in identical blood samples in patients with CRC. At the time of clinical presentation, proteases are more sensitive indicators of diagnosis than the most commonly used markers CEA and CA 19-9. Even though the benefits of multiparametric tumor markers analyses are highly questionable, the levels of sensitivity and specificity reached in our study seem to open the door to such an approach in CRC. On the other hand, we demonstrate the clear-cut prognostic impact of serum CATB, CATL and plasma PAI-1 antigen levels for patients with CRC. Moreover, in multivariate analysis, PAI-1 and CATB were strong and independent prognostic factors in CRC. Finally, our results suggest that CATB, CATL and PAI-1 may have a crucial role not only in the invasive process of cancer, but also in the progression of colorectal precancerous lesions into cancer.

## Abbreviations

AUC: area under curve; CATB: cathepsin B; CATL: cathepsin L; CEA: carcinoembryonic antigen; CA 19-9: carbohydrate antigen 19-9; 95%CL: 95% confidence limits; ELISA: enzyme-linked immunoassay; HR: Hazard ratio; PAI-1: plasminogen activator inhibitor type-1; pro-uPA: pro-urokinase-type plasminogen activator; ROC: receiver operating characteristics; rS: Spearman rank correlation coefficient; uPA: urokinase-type plasminogen activator.

## Competing interests

The authors declare that they have no competing interests. There is no conflict of interest, financially or personally, with other people or organization that could inappropriately influence our work.

## Authors' contributions

LH had the initial idea for the study, participated in the study design of the study, performed statistical analysis, generated experimental data and drafted the manuscript. FF participated in the review and commentary of documents relative to the study and manuscript editing. RC, GI, MDP and MP generated experimental data. LDM performed statistical analysis. IH performed statistical analysis and drafted the manuscript. ZT participated in the design and coordination of the study. Each author participated in the study to a significant extent. All authors read and approved the final manuscript.

## Pre-publication history

The pre-publication history for this paper can be accessed here:


